# Focusing on *Helicobacter pylori* infection in the elderly

**DOI:** 10.3389/fcimb.2023.1121947

**Published:** 2023-03-10

**Authors:** Hang Gong, Hui-Mei Xu, De-Kui Zhang

**Affiliations:** Department of Gastroenterology, Lanzhou University Second Hospital, Lanzhou, Gansu, China

**Keywords:** *Helicobacter pylori*, eradication therapy, elderly, side effect, drug resistance

## Abstract

As a confirmed carcinogen, Helicobacter pylori (*H. pylori*) is the main cause of inflammatory diseases of the upper digestive tract and even gastric cancer. There is a high prevalence of *H. pylori* infection among the elderly population, which may cause adverse clinical outcomes. Particularly noteworthy is that guidelines or expert consensus presently available on *H. pylori* infection overlook the management of the elderly population as a special group. A brief overview of *H. pylori* in the elderly is as follows. The detection of *H. pylori* infection can be divided into invasive and non-invasive techniques, and each technique has its advantages and shortcomings. There may be more side effects associated with eradication treatment in elderly individuals, especially for the frail population. Physical conditions and risk-benefit assessments of the elderly should be considered when selecting therapeutic strategies for *H. pylori* eradication. Unless there are competing factors, elderly patients should receive *H. pylori* eradication regimens to finally reduce the formation of gastric cancer. In this review, we summarize the latest understanding of *H. pylori* in the elderly population to provide effective managements and treatment measures.

## 1 Introduction

The challenge of population aging is becoming increasingly intense in several developed and developing countries, such as China, Japan, and the United States (Zhao et al., 2019). An Italian study showed that about 40% of more than 3,000 people over 60 years old had gastrointestinal symptoms due to the presence of disordered digestive function in the elderly population (Pilotto et al., 2011). The functional decline of the upper gastrointestinal tract is mainly characterized by the atrophic gastric mucosa and reduced digestive enzyme activity, and infection [e.g., Helicobacter pylori (*H. pylori*)], non-steroidal anti-inflammatory drugs (NSAIDs) and other factors can lead to the further deterioration (Huang et al., 2021a).

Higher prevalence and prolonged accumulation of *H. pylori* infection in the elderly lead more easily to atrophic gastritis, intestinal metaplasia, and even gastric cancer (Weck and Brenner, 2006; Weck et al., 2007; Toyokawa et al., 2010). Due to a decline in physical function from underlying diseases, complications of some diseases like renal insufficiency and more severe drug adverse effects, different eradication therapy regimens, and ideal drug dosing, especially antibiotics, are still not well defined for the elderly (Zendehdel and Roham, 2020). It is well known that currently updated guidelines or expert consensus on *H. pylori* infection are either unmentioned or understated about assessments of risks and benefits, therapeutic strategies, and treatment-related adverse effects among the elderly (Sugano et al., 2015; Liu W. Z. et al., 2018; Malfertheiner et al., 2022). As can be seen, the concern about *H. pylori* infection among older adults is still relatively low. In this review we presented the most recent advancements in *H. pylori* infection in the elderly, involving several aspects, such as epidemiology, diagnosis, treatment, and adverse events.

## 2 Epidemiology

It is estimated that approximately 50% of the world’s population is infected with *H. pylori* usually acquired in childhood (Suerbaum and Michetti, 2002). The infection rate of *H. pylori* in the elderly having higher education, dominated by significant mental work or living in more economically developed regions is relatively lower (Nurgalieva et al., 2002; Matsuhisa et al., 2015). The correlation between some living habits (e.g., tea drinking, smoking, alcohol intake) and *H. pylori* infection is uncertain. An early epidemiological survey showed the prevalence of *H. pylori* infection among the elderly was 83.4% (84.7% in males and 82.1% in females) in Beijing, China (Zhang et al., 2005). However, the *H. pylori* infection rate in Beijing was 46.5% (507/1090) from a recent investigation, with a significantly higher rate in males than in females (51.8% vs 42.5%). And, the total infection rate increased gradually with age (Zhu et al., 2020). Several early studies in other countries revealed that the infection rate of *H. pylori* in the elderly with peptic ulcer disease is 58%-78%, but only 40% to 56% of whom were tested for *H. pylori* infection, and 50% to 73% of whom with a positive test were subsequently treated with antibiotics (Roll et al., 1997; Ofman et al., 2000; Pilotto, 2001). Studies also confirmed the infection was more common in the elderly population, with a prevalence ranging from 68% to 86.5% in hospitalized patients, most of whom came from nursing homes (Pilotto et al., 1996; Regev et al., 1999). However, there is updated evidence of a descending trend in *H. pylori* infection rate in both adults and children from some countries (Bures et al., 2012; Tonkic et al., 2012).

Concerning antibiotic resistance, a recent study revealed that the overall resistance rates of clarithromycin, levofloxacin and metronidazole in the Chinese population were 17.76%, 19.66% and 95.5%, respectively, and the resistance rates of clarithromycin and levofloxacin were highest in the elderly (Ji et al., 2016). Similarly, another study showed that middle-aged and elderly patients exhibited higher resistance rates to clarithromycin, azithromycin, levofloxacin, and moxifloxacin compared to patients younger than 40 years old (Liu D. S. et al., 2018). The infection continues if left untreated and this cumulative effect is even more pronounced in older people. Taken together, drug resistance and high prevalence of *H. pylori* infection among elderly patients characterize its epidemiology. Thus, an increased focus is needed on diagnosing and treating *H. pylori* infection in the elderly.

## 3 H. pylori assays

Modalities for evaluating the *H. pylori* infection can be divided into invasive and non-invasive tests (Yilmaz et al., 2006). Invasive methods for directly detecting *H. pylori* in biopsy samples during endoscopy involve rapid urease test (RUT), histology and bacterial culture (Kim S. E. et al., 2020). The C-urea breath test (UBT), serological blood test, and stool antigen test (SAT) are the most commonly used non-invasive tests (Vonkeman et al., 2012). There are advantages, disadvantages, and limitations to each approach.

### 3.1 Non-invasive tests

The UBT still remains the gold standard in non-invasive methods dependent of urease activity of *H. pylori* (Toyoshima et al., 2018). In comparison with ^14^C-UBT, ^13^C-UBT most widely used in the elderly is a stable isotope with high safety and no radioactivity hazards (Xie et al., 2020). The greatest advantage of UBT makes it possible to take samples throughout the entire stomach and avoids the appearance of false negatives upon focal distribution of *H. pylori* in the stomach. Studies demonstrated that ^13^C-UBT had a sensitivity of 100%, a specificity of 95.7%, an accuracy of 98% for the diagnosis of *H. pylori* in the elderly, and 14C-UBT obtained a sensitivity of 91.4%, a specificity of 93.8% for the elderly (Pilotto et al., 2000b; Atli et al., 2012). However, the sensitivity and accuracy of ^13^C-UBT for elderly participants are lower than those of non-elderly patients (Choi et al., 2011). As can be seen that UBT is an accurate, practical and convenient test method (Gisbert and Pajares, 2005). Eisdorfer I et al. (Eisdorfer et al., 2018) indicated that the average UBT values increased significantly with age (28.6‰ in young group and 32.3‰ in elderly group). The possible explanations are not only the high *H. pylori* load or density in the elderly but also are reduced basal metabolic rate or impaired gastric emptying (Liu et al., 1995; Chang et al., 2002; Roberts and Dallal, 2005; Perets et al., 2019). Although a uniform cut-off value of UBT for all age groups is conducted by manufacturers, a higher threshold value of UBT might be recommended to determine the presence of *H. pylori* infection in the elderly population (Perets et al., 2019).

Based on the immune system triggered by *H. pylori* infection, the serological blood test is used to detect specific antibodies, with the 74.4% sensitivity, 59% specificity and 67% diagnostic accuracy in the elderly (Pilotto et al., 1996). In the participants over 60 years, the specificity of the serological blood test is lower than in those < 40 years (Choi et al., 2011). *H. pylori* antibodies can still remain positive six months after eradication due to the possibility of antibodies persisting in the blood for a prolonged period. Positive antibody test results cannot distinguish the presence of *H. pylori* current infection, so this method is mainly used for epidemiological investigation, rather than for follow-up after eradication (Koyama et al., 2016). False-negative serological results may occur in elderly patients with immune deficiency or protein malnutrition due to the lack of antibody response (Cizginer et al., 2014). In fact, it can also be used to observe the former presence of *H. pylori* in the elderly with atrophic gastritis.

*H. pylori*, which is excreted with the stool following renewal and shedding of gastric mucosa epithelial cells, can be confirmed by detection of fecal antigens. SAT remains unaffected by atrophic gastritis, ulcer or intestinal metaplasia in the elderly population. It is also applicable to detect *H. pylori* after gastrectomy. The UBT tends to appear false negative results, whereas SAT is not affected by decreased gastric acid secretion of remnant stomach upon reduced gastral cavity (Best et al., 2018). The diagnostic accuracy of SAT is similar to that of UBT according to the Maastricht IV/Florence consensus report (Malfertheiner et al., 2012). Several studies suggested SAT showed high specificity and accuracy for the elderly, but its sensitivity remained unsatisfactory (Inelmen et al., 2004; Inelmen et al., 2005; Han et al., 2020). Relatively, SAT appears to have a higher sensitivity for the diagnosis of individuals younger than 60 years than elderly individuals aged 60 and over, suggesting a slight tendency for decreasing sensitivity with increasing age (Choi et al., 2011). This might be because constipation commonly occurs among the elderly population, which contributes to longer *H. pylori* transport time in the intestinal tract so as to lead to degradation of bacterial antigens (Salles-Montaudon et al., 2002). In addition, SAT appears to be more advantageous in the elderly reluctant to receive the UBT or with suboptimal breathing cooperation (e.g., severe pulmonary fibrosis and chronic obstructive pulmonary disease) (Konstantopoulos et al., 2001; Alzoubi et al., 2020).

### 3.2 Invasive tests

The main strength of the invasive methods is a lesion can be identified in the stomach by a gastroscopy examination, especially in the context of increased risk of gastric cancer and gastric mucosa with dysplasia in the elderly. Endoscopy-based *H. pylori* detection method should be preferred in the elderly with alarm symptoms (e.g., emaciation, anemia) to avoid missing important diseases (Zendehdel and Roham, 2020). The RUT with several advantages of rapidness, convenience and high accuracy can be used as a rapid detection method of *H. pylori* in elderly patients undergoing gastroscopy (Uotani and Graham, 2015). In spite of its high specificity and accuracy, the RUT is considered to have a lower sensitivity in older patients compared to younger patients (57% vs 75%) (Rogge et al., 1995; Abdalla et al., 1998). The possible reason is that *H. pylori* is focally distributed within the stomach and false negative results may occur when the bacterial count in the biopsy specimen is less than 1 × 104 (Godbole et al., 2020). Multi-point biopsy in the stomach can improve the positive rate (Hsu et al., 2010). The RUT is also not recommended for follow-up after *H. pylori* eradication (Malfertheiner et al., 2017).

Histology is effective for evaluating *H. pylori*-associated gastritis (Malfertheiner et al., 2017). Choi J et al. (Choi et al., 2011) showed that the sensitivity, specificity, and accuracy of histology for the diagnosis of *H. pylori* in elderly participants were lower than those 40-60 years and those under 40 years. Moreover, according to the Sydney system for histological diagnosis and classification of chronic gastritis, objective evaluation indices for *H. pylori* are taken into account. The tissue biopsy and histological staining are recommended for elderly patients with suspected lesions upon gastroscopy to determine the presence of cancerous lesions and/or *H. pylori* infection (Zhao and Chi, 2022). The most conventional staining is hematoxylin-eosin staining, which can be false negative for *H. pylori* when the specimen contains little bacterial count (Wang et al., 2010). Notably, special stainings with high contrast (e.g., Giemsa staining, immunohistochemical staining) contribute to diagnose *H. pylori* infection (Wang et al., 2010; Batts et al., 2013; Kocsmar et al., 2017).

**Figure 1:**
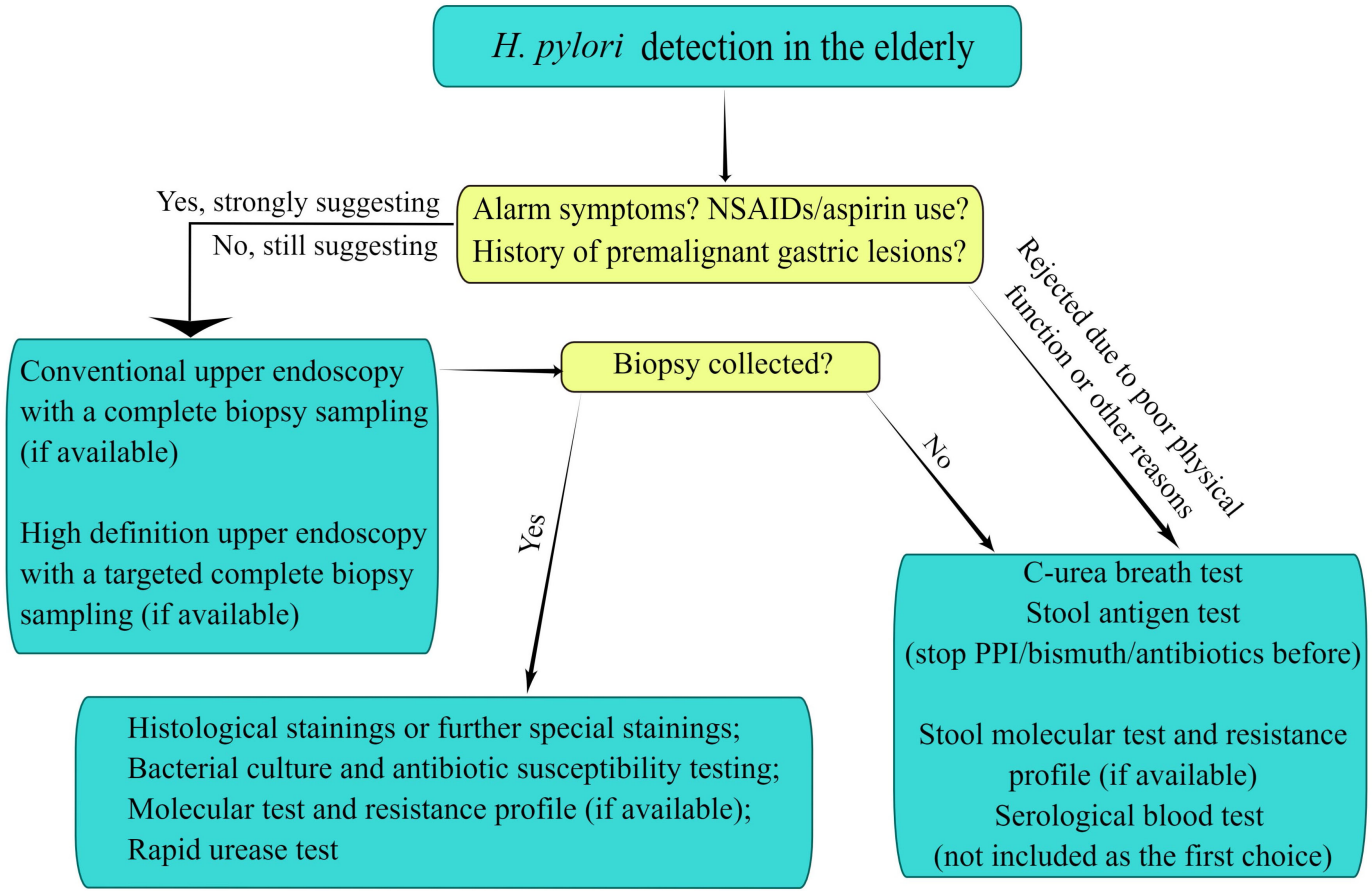
Diagnostic evaluation to detect Helicobacter pylori infection in the elderly. The figure is drawn by Figdraw.

*H. pylori* is a microaerophilic bacterium and its isolation culture depends on stringent culture conditions. The positive rate of cultures is affected by improper storage and transport of specimens, as well as too low concentrations of inoculum and so on (Leszczynska et al., 2010; Gong et al., 2015). Thus, the bacterial culture is mainly used for the epidemiological investigation and antimicrobial susceptibility testing in the resistant elderly population where *H. pylori* eradication failed (Rojas-Rengifo et al., 2019). *H. pylori* culture taken from gastric mucosal tissue samples under appropriate conditions has a satisfactory specificity but a somewhat lower sensitivity (70%-90%) (Wang et al., 2015). Moreover, the molecular biology technique is not a conventional method for the diagnosis of *H. pylori* infection (Liu W. Z. et al., 2018), but it with high sensitivity and specificity is superior to bacterial culture and susceptibility testing, which has important clinical value for guiding the choices of antibiotics in the elderly (**Figure 1**) (Liu W. Z. et al., 2018; Marrero et al., 2021).

## 4 Clinical features

H. *pylori* infection in the elderly induces the inflammatory response of gastric mucosa, abnormal secretion of gastrointestinal hormones and gastric acid to affect gastroduodenal motility and sensitivity, which is associated with dyspeptic symptoms (Suzuki and Moayyedi, 2013). *H. pylori* infection is the main factor of peptic ulcer, which can be promoted to heal, with a decrease in the relapse rate by eradicating *H. pylori* (Gisbert et al., 2012). Moreover, benign diseases of the digestive system associated with *H. pylori* infection in the elderly also include functional dyspepsia, non-atrophic gastritis, gastroesophageal reflux disease, and even chronic atrophic gastritis (Pilotto and Franceschi, 2014). Gastric mucosa-associated lymphoid tissue lymphoma (MALT) and gastric cancer are also associated with *H. pylori* infection in the elderly. Nagy P et al. (Nagy et al., 2016) demonstrated that 60%~80% of patients with *H. pylori*-positive locally gastric MALT achieved remission after eradicating *H. pylori*, suggesting that eradication of *H. pylori* is the first-line therapy for localized gastric MALT. Cancer of the stomach ranks fourth on the global cancer list, with the highest incidence in the elderly population (Zeng et al., 2020). *H. pylori* has been identified as a type I carcinogenic factor of gastric cancer, which is associated with 90% of non-cardia gastric cancer (Herrero et al., 2014; Wang et al., 2018). One study from Japan showed that 2.9% of the *H. pylori*-infected subjects developed gastric cancer following 7.8 years, whereas none of the non-infected subjects progressed to gastric cancer. Notably, histological findings characterized by severe gastric atrophy, intestinal metaplasia, and corpus-predominant gastritis contribute to the development of gastric cancer (Uemura et al., 2001).

*H. pylori* infection in the elderly also is associated with several extra-digestive diseases, such as iron-deficiency anemia (IDA), idiopathic thrombocytopenic purpura (ITP), vitamin B_12_ deficiency (Stasi et al., 2009; Yuan et al., 2010). A meta-analysis suggested that improved anemia and iron status could be attributable to the eradication of *H. pylori* in patients with IDA, especially in patients with moderate or severe anemia (Yuan et al., 2010). It has been reported that approximately 50% of *H. pylori*-positive ITP patients showed complete remission following the eradication of *H. pylori*, with improved platelet counts (Stasi et al., 2009). Vitamin B_12_ deficiency is common in the elderly, and may be related to gastrectomy, proton pump inhibitors (PPIs), *H. pylori* infection, and other factors. The eradication of *H. pylori* can only play an auxiliary role (Carmel et al., 2001). Besides the above-mentioned extra-gastric diseases, *H. pylori* infection has also been reported to be associated with cardiovascular diseases, Alzheimer’s disease, Parkinson’s disease, stroke, etc. (Liu W. Z. et al., 2018). However, these associations are still inconsistent and need to be further clarified.

## 5 Treatment

Due to a decrease in gastric mucosal barrier function in the elderly, *H. pylori* eradication is beneficial in remission of the disease and repairing the damaged mucosa (Cui et al., 2016). The incidence rates of chronic atrophic gastritis and intestinal metaplasia are higher in the elderly than in young and middle-aged people (Liu et al., 2019). A retrospective study enrolling 73237 patients confirmed that the cumulative incidence rate of gastric cancer decreased apparently after *H. pylori* eradication and the benefits were more marked in the elderly (Leung et al., 2018). Higher proportion of older people are often on acetyl salicylic acid (aspirin) and/or nonsteroidal anti-inflammatory drugs (NSAIDs) against cardiovascular disease and/or gout, and the clinical benefits are obvious by *H. pylori* eradication (Hernandez-Diaz and Garcia, 2006). Due to deteriorating physical condition, serious comorbidities and renal dysfunction, elderly patients are more likely to experience adverse drug reactions. Thus, a comprehensive risk-benefit assessment and individualized treatment should be performed to eradicate *H. pylori* in the elderly (Norgard et al., 2009; Liu W. Z. et al., 2018). In this paper, we compiled studies of different treatment regimens for *H. pylori* eradication in the elderly (**Table 1**).

**Table 1:** Studies of different treatment strategies and corresponding eradication rates.

Author (reference)	Age (years)	Treatment	Treatment duration	Eradication rate
Ang et al., 2022	≥60	Vonoprazan 20mg + amoxicillin 1g + clarithromycin 500mg twice/day	7 days	95.1%
	≥60	PPI + amoxicillin 1g + clarithromycin 500mg twice/day	14 days	95.5%
Gao and Fan, 2022	≥60	PPI + bismuth + two antibiotics selected from amoxicillin, clarithromycin, fluoroquinolone, furazolidone and tetracycline	14 days	ITT: 92.04% PP: 96.30%
Durazzo et al., 2021	≥65	Omeprazole + amoxicillin 1g+ clarithromycin +500mg twice daily	7, 10 or 14 days	ITT: 70.9% PP: 73.1%
Kim J. L. et al., 2020	≥65	Esomeprazole 40mg + amoxicillin 1g + clarithromycin 500mg twice/day	10 days	73.9%
	≥65	Esomeprazole 40mg + bismuth 300mg twice/day + metronidazole 500mg 3 times daily + tetracycline 500mg 4 times daily	10 days	93.8%
Gao et al., 2020	≥60	Rabeprazole 10 mg + amoxicillin 1g three times daily	14 days	90.5%
Kobayashi et al., 2019	65-74	PPI/vonoprazan + amoxicillin + clarithromycin	7 days	88.9%
	65-74	PPI/vonoprazan + amoxicillin + metronidazole	7 days	97.4%
	≥75	PPI/vonoprazan + amoxicillin + clarithromycin	7 days	87.8%
	≥75	PPI/vonoprazan + amoxicillin + metronidazole	7 days	87.5%
Zhang et al., 2017	≥60	Berberine hydrochloride 5 tablets + esomeprazole 1 tablet + amoxicillin 2 capsules + clarithromycin 1 tablet twice/day	14 days	86.6%
	≥60	Bismuth tartrate 4 tablets + esomeprazole 1 tablet + amoxicillin 2 capsules + clarithromycin 1 tablet twice/day	14 days	91.4%
Chuah et al., 2016	≥60	Esomeprazole 40mg + amoxicillin 1g twice/day + levofloxacin 500mg once/day	10 days	79.5%
	≥60	Esomeprazole 40mg + amoxicillin 1g twice/day (5 days) followed by esomeprazole 40mg + levofloxacin 500mg once/day+ metronidazole 500mg 3 times daily (5 days)	10 days	96.9%
Tsujimae et al., 2016	>70	Esomeprazole 20mg + clarithromycin 200mg + amoxicillin 750mg twice/day	7 days	84.0%
	>70	Vonoprazan 20mg + clarithromycin 200mg + amoxicillin 750mg twice/day	7 days	87.1%
Zhou et al., 2016	>60	PPI (rabeprazole 10mg or esomeprazole 20mg twice/day) + amoxicillin 1000mg two/three times daily+ clarithromycin 500mg twice/day or tinidazole 500mg three times daily	10 days	97.1%
	>60	Esomeprazole 20 mg + amoxicillin 1000 mg + clarithromycin 500 mg + bismuth potassium citrate 220 mg twice daily	10 days	77.1%
	>60	Esomeprazole 20 mg + amoxicillin 1 g + clarithromycin 500 mg + tinidazole 500 mg twice daily	10 days	93.5%
Heo et al., 2015	≥60	Esomeprazole 20 mg + amoxicillin 1 g + clarithromycin 500 mg + metronidazole 500 mg twice daily	10 days	92.5%
	≥60	Esomeprazole 20 mg + amoxicillin 1 g twice daily (5 days) followed by esomeprazole 20 mg + amoxicillin 1 g + clarithromycin 500 mg + metronidazole 500 mg twice daily (5 days)	10 days	87.1%
Tai et al., 2015	≥60	Esomeprazole 40 mg + amoxicillin 1 g + metronidazole 500 mg + clarithromycin 500 mg twice daily	7 days	92.8%
	≥60	Esomeprazole 40 mg + amoxicillin 1 g + clarithromycin 500 mg twice daily	7 days	75.0%
Oh et al., 2014	≥60	Rabeprazole 20 mg + amoxicillin 1g twice daily (7 days) followed by rabeprazole 20 mg + amoxicillin 1g + clarithromycin 500 mg + metronidazole 500 mg twice daily (7 days)	14 days	91.5%
	≥60	Rabeprazole 20 mg + amoxicillin 1g twice daily (7 days) followed by rabeprazole 20 mg + clarithromycin 500 mg + metronidazole 500 mg twice daily (7 days)	14 days	87.5%
Chen et al., 2014	≥60	Levofloxacin 500 mg + amoxicillin-clavulanate 875 mg/125 mg + rabeprazole 20 mg twice daily	7 days	ITT: 90.9% PP: 95.2%
	≥60	Clarithyromicin 500 mg + amoxicillin 1g + rabeprazole 20 mg twice daily	7 days	ITT: 42.3% PP: 42.3%
Heo et al., 2014	≥70	Lansoprazole 30 mg + amoxicillin 1g + clarithromycin 500 mg + metronidazole 500 mg twice daily	10 days	77.3%
	≥70	Lansoprazole 30 mg + amoxicillin 1g + clarithromycin 500 mg twice daily	10 days	72.4%
Lim et al., 2013	>70	Rabeprazole 20 mg + amoxicillin 1 g twice daily (7 days) followed by rabeprazole 20 mg + clarithromycin 500 mg + metronidazole 500 mg twice daily (7 days)	14 days	63.6%
	>70	Rabeprazole 20 mg + amoxicillin 1 g + clarithromycin 500 mg + metronidazole 500 mg twice daily	14 days	45.5%
Park et al., 2012	≥60	Rabeprazole 20 mg + amoxicillin 1 g twice daily (5 days) followed by rabeprazole 20 mg clarithromycin 500 mg + metronidazole 500 mg twice daily (5 days)	10 days	96.7%
	≥60	Rabeprazole 20 mg + amoxicillin 1 g + clarithromycin 500 mg twice daily	7 days	76.6%
Dore et al., 2006	≥65	Esomeprazole 20 mg + tetracycline 500 mg + metronidazole 500 mg + bismuth subcitrate tablets 240 mg twice daily	10 days	ITT: 91.0% PP: 95.0%
Zullo et al., 2005	≥65	Rabeprazole 20 mg + amoxicillin 1 g twice daily (5 days) followed by rabeprazole 20 mg + clarithromycin 500 mg + tinidazole 500 mg twice daily (5 days)	10 days	ITT: 94.4% PP: 96.6%
	≥65	Rabeprazole 20 mg + clarithromycin 500 mg + amoxicillin 1 g twice daily	7 days	ITT: 80.0% PP: 82.8%
Pilotto et al., 2001	≥60	Pantoprazole 40 mg once daily + amoxicillin 1 g + clarithromycin 250 mg twice daily	5 days	ITT: 83.0% PP: 94.0%
	≥60	Pantoprazole 40 mg once daily + amoxicillin 1 g + clarithromycin 500 mg twice daily	5 days	ITT: 79.0% PP: 88.0%
Pilotto et al., 2000a	≥60	Pantoprazole 40 mg once daily + amoxycillin 1 g + clarithromycin 250 mg twice daily	7 days	ITT: 85.7% PP: 88.5%

ITT, intention-to-treat; PP, per-protocol; PPI, proton pump inhibitor.

### 5.1 Vonoprazan- or proton pump inhibitor (PPI)-based triple therapy

The standard triple therapy containing a PPI and two antibiotics is widely accepted since the first Maastricht Consensus (Malfertheiner et al., 1997). Three early randomized controlled trials have shown that the standard triple regimen (pantoprazole/rabeprazole, amoxicillin and clarithromycin) had effective *H. pylori* eradication rates [intention-to-treat (ITT): 79.0%-85.7%; per-protocol (PP): 82.8%-94.0%] in the elderly aged over 60 (Pilotto et al., 2000a; Pilotto et al., 2001; Zullo et al., 2005). The efficacies of clarithromycin 250mg twice daily and 5-day regimen were comparable to 500mg twice daily and 7-day regimen, respectively, but the lower dose of clarithromycin 250mg twice daily and 5-day regimen minimized adverse events and costs (Pilotto et al., 2001; Zullo et al., 2005). However, pantoprazole-based triple therapy was less effective in the prevention of gastroduodenal injury in *H. pylori*-positive elderly patients taking NSAIDs than pantoprazole monotherapy for one month (Pilotto et al., 2000a). Multiple studies (Chen et al., 2014; Heo et al., 2014; Chuah et al., 2016; Durazzo et al., 2021; Ang et al., 2022; Park et al., 2012; Tai et al., 2015; Tsujimae et al., 2016; Zhou et al., 2016; Kobayashi et al., 2019; Kim J. L. et al., 2020) on *H. pylori* eradication have compared elderly individuals to those of non-elderly population, then from which we observe that the increase with age does not affect efficacy of some PPI-based triple therapies (involving amoxicillin + clarithromycin/metronidazole/levofloxacin, amoxicillin-clavulanate + levofloxacin) for *H. pylori* eradication. But from overall presented literature data (**Table 1**), the efficacy of PPI-based triple therapies in the elderly population remains controversial within the context of the higher resistance rates for antibiotics (e.g., clarithromycin, metronidazole and levofloxacin). Possibly, since differences are found in the antibiotic resistance rates from different regions, the eradication rate of *H. pylori* with PPI-based triple therapies shows obvious fluctuation (approximately 40%-95%) in the elderly, with the eradication rate lower in Chinese Taiwan and higher in Singapore and Japan (Chen et al., 2014; Heo et al., 2014; Chuah et al., 2016; Durazzo et al., 2021; Ang et al., 2022; Park et al., 2012; Tai et al., 2015; Tsujimae et al., 2016; Kobayashi et al., 2019; Kim J. L. et al., 2020).

Moreover, the eradication rate of clarithromycin resistance-guided (tailored) triple therapy is significantly higher than those of empirical triple plus bismuth therapy and concomitant therapy, and the efficacy of triple therapy containing amoxicillin-clavulanate also is more favorable, both for young and elderly people (Chen et al., 2014; Zhou et al., 2016). Interestingly, these eradication therapies are more effective among the elderly than in the young, when stratifying individuals by age. Clinicians are often reluctant to treat patients with advanced age, possibly due to unfounded concerns about adverse effects. However, Kobayashi S et al. (Kobayashi et al., 2019) showed that there was no significant difference in the eradication rate and the frequency of adverse event among the super-elderly (over age 75), elderly (aged 65-74) and younger groups (under age 65). The vonoprazan, as a new potassium-competitive acid blocker, produces a stronger and more lasting inhibitory effect of intragastric acid secretion than traditional PPIs (Kagami et al., 2016). Ang D et al. (Ang et al., 2022) demonstrated that 7-day vonoprazan-based triple therapy was as effective as 14-day PPI-based triple therapy, and old age did not influence the efficacies. Interestingly, Tsujimae M et al. (Tsujimae et al., 2016) further confirmed that the efficacy of vonoprazan-based triple regimen was superior to PPI-based triple regimen in patients younger than 70 upon the same timing of administration but both of regimens were as effective in patients aged over 70.

### 5.2 Triple plus bismuth therapy

Due to the increase of antimicrobial resistance globally, the bismuth quadruple therapy has been recommended as the empiric primary treatment regimen in “Fifth Chinese National Consensus Report on the management of Helicobacter pylori infection (Liu W. Z. et al., 2018)”. Tetracycline with the low resistance rate may exhibit higher eradication rate. A study from Italy showed tetracycline-containing bismuth quadruple therapy had an excellent *H. pylori* eradication rate of more than 90% in the elderly (Dore et al., 2006). Another study has shown that 14-day triple plus bismuth therapy was as well effective as triple plus berberine therapy for both the elderly and the young. Similarly, old age did not influence the efficacies (Zhang et al., 2017). But the study by Zhou L et al. showed that the eradication rate of 10-day triple plus bismuth therapy was less efficient than concomitant therapy (77.1% vs 93.5%) for the elderly in a setting with higher rates of resistance to clarithromycin and metronidazole (Zhou et al., 2016). Thus, clarithromycin resistance-guided bismuth quadruple therapy (tailored therapy) could be a good alternative to improve eradication efficacy compared to empiric treatment (Gao and Fan, 2022; Kim J. L. et al., 2020). Moreover, given not drug-resistant bismuth, high safety with the short-term application of bismuth, and wide antibiotic selections after eradication failure, bismuth quadruple therapy appears to be more advantageous for first eradication treatment than non-bismuth quadruple therapy (Ford et al., 2008; Dore et al., 2016). In short, the combination of bismuth and the triple therapy improves the eradication rate of *H. pylori* in the elderly, also dependent of the cure rate of the resistant strain and the resistance rate from endemic areas (Dore et al., 2016). If a high rate of resistance exists, triple plus bismuth therapy cannot achieve the ideal eradication rate, which needs to switch to a non-bismuth regimen or susceptible antibiotic to eradicate *H. pylori* (Malfertheiner et al., 2012).

### 5.3 Sequential and hybrid therapies

Sequential and hybrid therapies may be more effective for the eradication of *H. pylori*. Several studies have shown that the efficacy of sequential therapy with eradication rate higher than 90% was superior to standard triple therapy in the elderly (Zullo et al., 2005; Park et al., 2012; Chuah et al., 2016). Moreover, hybrid therapy also exhibits similar excellent efficacy to sequential therapy (Oh et al., 2014; Heo et al., 2015). Overall, we observe in these studies that the elderly does not affect the efficacies of sequential and hybrid therapies compared to the non-elderly. The mechanism by which sequential therapy and hybrid therapy exhibit an effective action is that the administration of amoxicillin within the initial 5 or 7 days of treatment may significantly reduce the density and number of resistant bacteria to increase the efficacy of subsequent clarithromycin (Gatta et al., 2009). Furthermore, the resistance to nitroimidazole does not seem to influence the eradication efficacy of non-bismuth quadruple therapy (Zullo et al., 2000; Treiber et al., 2002). However, one study from Korea showed suboptimal efficacy of sequential therapy in the elderly though significantly higher than standard triple therapy, which was probably related to high antibiotics resistance (Lim et al., 2013). The efficacy of sequential therapy may be reduced in the presence of single drug resistance to metronidazole or clarithromycin, so this regimen is less recommended in Chinese adults (Zhou et al., 2014; Liu W. Z. et al., 2018).

### 5.4 Concomitant therapy

Concomitant therapy shows shorter treatment duration and less complex drug administration as compared to sequential therapy and hybrid therapy. Practically, this can make treatment more convenient and improve patient compliance and physician preferences towards healthcare (Hsu et al., 2014). Heo J et al. (Heo et al., 2015) confirmed that concomitant therapy has similar efficacy compared to hybrid therapy in the elderly, and their efficacies did not decrease with the age. When bismuth quadruple therapy is not available, it is recommended to administer concomitant therapy in high-prevalence areas of clarithromycin resistance by the Maastricht IV/Florence Consensus Report (Malfertheiner et al., 2012). Several studies have confirmed that concomitant therapy exhibited excellent *H. pylori* eradication rates regardless of old age (Heo et al., 2015; Tai et al., 2015; Zhou et al., 2016). And, two studies from Chinese mainland and Chinese Taiwan suggested that the efficacy of concomitant therapy was superior to standard triple therapy and bismuth quadruple therapy in the elderly (Tai et al., 2015; Zhou et al., 2016). However, one study from Korea showed that older patients aged over 70 had a significantly lower eradication rate than patients younger than 70 (45.5% vs 86.6%) (Lim et al., 2013). Considering too few subjects older than 70 years, this result remains questionable. A Randomized controlled trial with larger sample size is needed to investigate the effect of old age on the efficacy of concomitant therapy. When the high prevalence of dual resistance to clarithromycin and metronidazole is anticipated, concomitant therapy is actually double therapy consisted of PPI + amoxicillin and also difficult to eradicate *H. pylori* (Graham and Dore, 2016; Zhou et al., 2016).

### 5.5 High-dose dual therapy

High-dose PPI plus amoxicillin has gained much attention due to the low amoxicillin resistance and simple composition of drug. High-dose dual therapy as salvage therapy offers better efficacy as compared with bismuth quadruple therapy or triple therapy (Miehlke et al., 2006; Yang et al., 2015). Potent inhibitory gastric acid secretion and sufficient amoxicillin dosage may be the effective factors. High-dose dual therapy appears to be equally effective with current mainstream regimens for the eradication of *H. pylori* based on a meta-analysis (C. P. Gao et al., 2020). However, this effectiveness has rarely been validated in the elderly. Although the daily dose of dual therapy exhibits pronounced increase but it remains within the recommended safe range. Due to the reduced use of antibiotics and bismuth, the incidence of side effects is significantly reduced (Huang et al., 2021b). But this regimen is conducted in only the elderly patients without penicillin allergy and renal insufficiency. Gao W et al. (W. Gao et al., 2020) showed that the eradication rate of high-dose rabeprazole plus amoxicillin was as high as 90.5% and 91.5% in elderly and non-elderly patients with or without multiple complications, respectively. Therefore, high-dose PPI plus amoxicillin may be a more likely alternative regimen for eradication of *H. pylori*.

## 6 Clinical outcomes

Since the researchers give little importance to *H. pylori* infection in the elderly, few studies for adverse events and/or adherence are available for analysis. Based on the available literature data, the incidence of adverse events with triple therapy in the elderly is approximately 10% or lower, which suggests that the triple therapy for the eradication of *H. pylori* in elderly patients is safe and well tolerated (Pilotto et al., 2001; Zullo et al., 2005; Kobayashi et al., 2019). Adverse events mainly include diarrhea, abdominal pain, vomiting, urticaria and so on. Diarrhea predominates among them and side effects are rarely a reason for patients to discontinue treatment. Pilotto A et al. (Pilotto et al., 2001) showed that older women were more susceptible to therapeutic side effects than older men. Moreover, Kobayashi S et al. (Kobayashi et al., 2019) indicted that there was no significant difference in the incidence of adverse events among younger, elderly and super-elderly patients. That said, increasing age did not seem to affect the risk of adverse events in terms of triple therapy.

Concerning triple plus bismuth therapy, adverse events are more common among the elderly. The major cause is bismuth-induced black stool, which is not clinically significant. Furthermore, other common adverse events include dysgeusia and diarrhea, and no one withdraws because of severe side effects according to the study by Gao C et al. (Gao and Fan, 2022). Only constipation is more prevalent among elderly patients than younger patients (5.6% vs 1.2%) (Gao and Fan, 2022). Another Italian study suggested that 23.7% of elderly patients receiving tetracycline-containing bismuth quadruple therapy complained of side effects without affecting their daily life, but another 9.7% of patients discontinued treatment due to severe side effects or subjective willingness (Dore et al., 2006). Interestingly, 23.7% of elderly patients reported significant improvements in bowel symptoms such as abdominal bloating and abnormal stool consistency. Regarding high-dose PPI plus amoxicillin therapy, one recent study showed the incidence of adverse events in non-elderly and elderly people was 13.4% and 9.5%, respectively, and 5.2% of elderly people and 7.3% of non-elderly people discontinued treatment due to adverse events (W. Gao et al., 2020). Symptoms such as rash, abdominal pain, and diarrhea were the most common adverse events and disappeared upon stopping treatment. Furthermore, Pea F et al. (Pea, 2015) showed that serious adverse events are most likely to appear in the frail elderly population due to drug-drug interactions and/or comorbidities. But antibiotics were used for the treatment of a broad spectrum of diseases and were not validated for the treatment for *H. pylori* in their study (Pea, 2015).

## 7 Challenges and future perspectives

The elderly is prone to antibiotic resistance due to previous overuse of antibiotics and long-term accumulation (Nguyen et al., 2019). In the elderly population with chronic respiratory or urinary tract infection, (fluoro)quinolone antibiotics are more commonly prescribed (Tandan et al., 2020). Lyu T et al. (Lyu et al., 2020) showed that middle-aged and elderly patients had higher secondary resistance rates to metronidazole and levofloxacin in southern China. It is suggested to conduct drug susceptibility testing for *H. pylori* in the elderly population to select appropriate antibiotics. With increasing age, the decrease in liver and kidney functions in the elderly contributes to the development of adverse drug reactions related to the mitigation of drug metabolism. Moreover, the decline in the function of the gastric mucosa defense system in the elderly leads to increased sensitivity to various damages and more noticeable drug reactions. Drug-drug interactions would trigger severe side effects in the elderly with multiple comorbidities (Tomita et al., 2019). Commonly used PPIs (for example, omeprazole) can interact with clopidogrel used in cardiovascular diseases; antibiotics (amoxicillin, clarithromycin, etc) for *H. pylori* can also interact with cardiovascular medications (such as statins, warfarin, etc) (Abrignani et al., 2021; Anrys et al., 2021). These factors mentioned above may result in elderly individuals not adhering to their dosage regimens. The elderly patients with frequent use of aspirin/NSAIDs or malabsorption of vitamin B12 also are considered candidates for *H. pylori* eradication (Malfertheiner et al., 2017). An individualized risk-benefit assessment should be undertaken in the elderly with the different physical conditions and benefits of eradication of *H. pylori*. Patients are subject to alterations of the intestinal flora in a short time due to *H. pylori* eradication, when necessary, supplementary microbial agents can be administered during or after *H. pylori* eradication to reduce gastrointestinal adverse effects (Malaty et al., 1999; Oh et al., 2016). More convenient regimens containing fewer drugs can increase medication adherence and reduce adverse reaction in the elderly. The vonoprazan-based or tetracycline-containing bismuth quadruple therapies, antibiotic resistance-guided regimens, and even currently hot high-dose dual therapy provide a new direction for the eradication of *H. pylori* in the elderly. The effectiveness and practicability of these regimens for the elderly merit further concern and investigation in the future.

## 8 Conclusions

**Figure 2:**
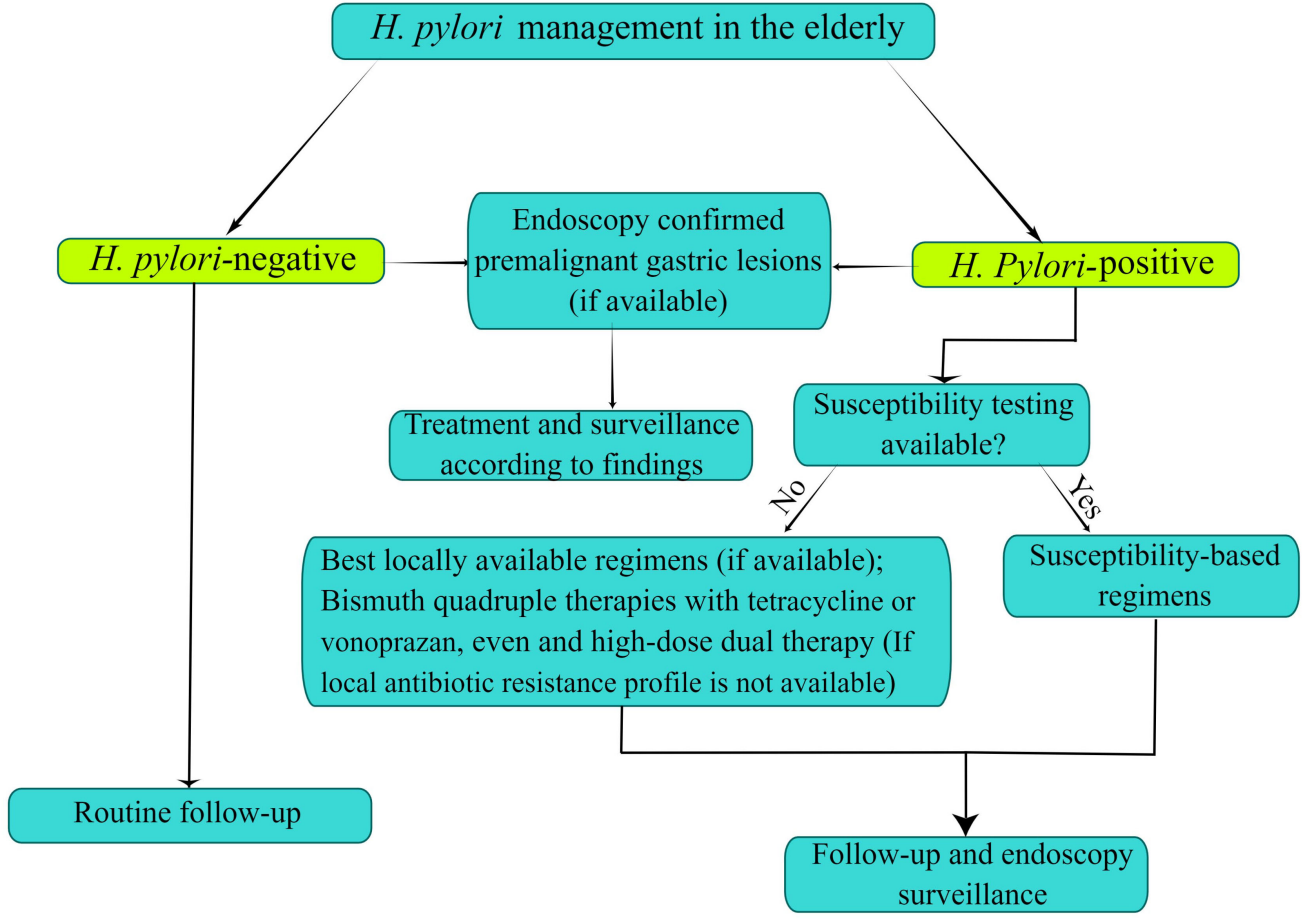
Flowchart of Helicobacter pylori infection control and management in the elderly. The figure is drawn by Figdraw.

As is well known, guidelines or expert consensus presently available on *H. pylori* infection overlook the management of the elderly population as a special group. We discussed the recent advances of *H. pylori* in the elderly population *via* epidemiology, diagnosis and treatment, etc. In summary, the choice of treatment strategies of *H. pylori* infection should be combined with the individualized characteristics and risk-benefit assessment of the elderly. To decrease the risk of gastric cancer, older patients should be received *H. pylori* eradication therapy unless with the presence of competing factors. There is an urgent need to explore effective treatment options due to the decreasing efficacy of triple therapy. Once failure treatment occurs in non-bismuth quadruple therapies, the choice of antibiotics will be confined. Thus, given the superiority of bismuth in quadruple therapies, bismuth quadruple therapy may be considered the empirical and preferable treatment regimen for *H. pylori* eradication in the elderly. If the antibiotic susceptibility testing against *H. pylori* is performed, the elderly patient should receive susceptibility-based regimens; if there is no susceptibility testing, elderly patients should be treated with the best locally available regimens (if available), and vonoprazan-based or tetracycline-containing bismuth quadruple therapies (if local antibiotic resistance profile is not available). Moreover, the currently hot high-dose dual therapy provides a new direction for the eradication of *H. pylori* in the future. At the same time, we strongly recommend conventional or high-definition upper endoscopy with a complete biopsy sampling for elderly patients with alarming symptoms (**Figure 2**). More details of the management of *H. pylori* infection in the elderly need to be further studied.

## Author contributions

HG: Writing-original draft. H-MX: Designing some contents of the manuscript. D-KZ: Writing and revising the manuscript. All authors contributed to the article and approved the submitted version.
